# Analysis of 10 years of open, laparoscopic, and robotic rectal surgeries in the community setting

**DOI:** 10.1016/j.sopen.2023.10.011

**Published:** 2023-11-01

**Authors:** Laura E. Cooper, Lena Morant, Maribeth Anderson, McKenzie Bedra, Cherif N. Boutros

**Affiliations:** a Department of Surgery, University of Maryland Medical Center, 22 S. Greene Street, Baltimore, MD 21201, United States of America; b Department of Surgery, University of Maryland Baltimore Washington Medical Center, 305 Hospital Drive, Tate Center, Suite 304, Glen Burnie, MD 21061, United States of America

**Keywords:** Colorectal, Minimally invasive surgery, Laparoscopic surgery, Robotic surgery, Rectal cancer, Community

## Abstract

**Background:**

Colorectal cancer is the fourth most common cancer in the US. Many of these patients will require operations. Although there is significant data in the literature that supports minimally invasive colorectal operations in the academic setting, few studies have examined their performance in community hospitals.

**Methods:**

Data was collected from a high-volume, university-affiliated, community center. Our Cancer Registry Database was queried to include any patients that had rectal surgery at our institution from 2010 to 2020. One hundred-twenty-two patients were identified and reviewed retrospectively. Main outcome measures include estimated blood loss (EBL), blood transfusion, time to first bowel movement, oncologic resection, length of stay (LOS), survival, and cost analysis.

**Results:**

Both robotic and laparoscopic operations resulted in lower average EBL, less blood transfusions, and less time to first bowel movement (*p* = 0.003, 0.006, 0.003, respectively). There was no significant difference in ability to achieve R0 resection, adequate lymph node retrieval, and adequate total mesorectal excision (TME, *p* = 0.856, 0.489, 0.500, respectively). LOS was significantly shorter for minimally invasive operations, 4.35 vs 8.48 days, and average survival was longest for laparoscopic operations at 7.19 years as compared to 5.55 years for open operations (*p* < 0.001, 0.026, respectively). Cost was lowest for robotic operations (0.003).

**Conclusions:**

Minimally invasive rectal operations, especially robotic, lead to better short- and long-term outcomes, equivalent oncologic resection, and are more cost-effective as compared to open operations even in the community setting, supporting continued performance and growth of robotic colorectal operations in the community setting.

**Key message:**

Although there is significant data in the literature that supports minimally invasive colorectal operations in the academic setting, few studies have examined their performance in community hospitals as this study does. This study found that minimally invasive rectal operations, especially robotic, lead to better short- and long-term outcomes, equivalent oncologic resection, and are more cost-effective as compared to open operations even in the community setting, supporting continued performance and growth of robotic colorectal operations in the community setting.

## Highlights

• Minimally invasive (MI) rectal operations had better short-term outcomes than open.
• No significant difference in adequate oncologic resection was found.
• MI operations had shortest length of stay, and robotic had the lowest cost.
• Average survival was longest for laparoscopic operations.

## Introduction

Colon and rectal cancers are the fourth most common cancer in the US with an estimated 104,610 new cases of colon cancer and 43,340 new cases of rectal cancer diagnosed in 2020 resulting in over 50,000 deaths [1]. Notably, about 1 in 23 men (4.4 %) and 1 in 25 women (4.1 %) will receive a diagnosis of colorectal cancer during their lifetime. Many of these patients will require operations which are performed in both the community and academic settings, and many are completed in a minimally invasive (MI) fashion. Although there is significant data in the literature that supports MI colorectal operations in the academic setting, few studies have examined their performance in community hospitals. Our hypothesis is that colon and rectal operations performed in the community can be performed minimally invasively at the same level as at academic institutions, thus adding to the current body of literature, mostly from academic institutions, supporting minimally invasive operations.

The challenges of rectal surgeries, especially due to anatomy and need for oncologic resection, have made MI operations appealing to the colorectal surgeon. Several studies have shown no statistically significant differences in pathologic outcomes, such as resection margins, lymph node (LN) retrieval, and TME quality, between MI and open operations [2,3]. Initially, laparoscopy was seen as a huge advantage in rectal operations given improved access to the limited space in the pelvis. However, since the advent of robotic surgery, the ideal operation for rectal cancer in particular has come into question. Uniquely, the robotic system allows for stable, three-dimensional views, improved dexterity, and better ergonomics, leading to its growing popularity in the field of colorectal surgery (CRS, [4]).

Although the literature is mixed on the benefits of robotic surgery, the overwhelming majority support its use in CRS. Robotic operations have shown lower rates of conversion to open surgery, shorter length of stay (LOS), less blood loss and reduced complication rates [[5], [6], [7], [8], [9]]. Conversely, many studies have noted that cost was significantly higher in robotic groups as compared to laparoscopic [[9], [10], [11]].

Notably, the majority of this data is from academic institutions. However, a fair number of CRS is performed in the community setting. For this reason, our study provides an important contribution to the current literature by highlighting the performance of MI and open colorectal operations in the community setting.

## Materials & methods

### Study subjects and design

This is a single-center, retrospective, case-control study comparing robot-assisted, laparoscopic, and open surgery in patients with rectal cancer from a high-volume community center: University of Maryland Baltimore Washington Medical Center (UM BWMC) in Glen Burnie, Maryland. A query was run by our Cancer Registry Database to include any patients that had rectal surgery at UM BWMC from January 1, 2010 to June 7, 2020. Patients with rectal cancer who underwent surgery within this timeframe were identified and reviewed retrospectively to obtain data on outcomes of the three different surgical approaches. There was no treatment or subject randomization as this was a chart review of pre-existing data already in the medical record. Patient selection was based on surgeon and patient preference. Notably, laparoscopy was first introduced in 2010 and robotic operations first introduced in 2011 – at the start of the timeframe for this study. Over time, minimally invasive operations grew in number. Specifically, >80 % of robotic operations took place during the last 5 years of the study time. A total of 122 patients underwent operations by 6 different surgeons and were analyzed. There was no exclusion of a specific population, sub-group or class. This study obtained institutional review board approval from the Human Research Protection Office (HP-00091709).

### Statistical analysis

IBM SPSS Statistics 26 and Microsoft Excel 2010™ were used for the statistical analysis. Quality data and cost analysis were tested using ANOVA. The χ^2^ test was used for categorical variables. Patient survival was calculated using the Kaplan Meier method.

## Results

### Demographics

During the selected time period, 28 patients underwent open surgery, 53 underwent laparoscopic surgery, and 41 underwent robotic surgery. There were no differences between groups in demographic data: sex, age at diagnosis, race, BMI, and insurance (Table 1).

**Table 1:** Demographic characteristics of patients.

Characteristic	Surgical approach			Totals	Significance
	Open (reference)	Laparoscopic	Robotic	Total	*p*-Value
Sex					0.965
Male	15	30	23	68	
Female	13	23	18	54	
Race					0.33
White	22	45	33	100	
Black	4	4	6	14	
Asian	0	1	2	3	
Other	2	3	0	5	
Insurance					0.089
Private	15	21	22	58	
Medicaid	0	3	0	3	
Medicare w Medicaid eligibility	0	2	1	3	
Medicare w/o supplement	3	13	3	19	
Medicare + supplement	3	5	6	14	
Other/unknown	3	2	8	13	
Uninsured	0	1	0	1	
Average BMI	26.52	28.57	28.54	27.87	0.496
Average age at diagnosis	61	60.25	59.71	60.32	0.86

### Postoperative outcomes

The differences in estimated blood loss (EBL), blood transfusion, and days until first bowel movement were calculated for open as compared to laparoscopic, open as compared to robotic, and open as compared to minimally invasive (MI, laparoscopic and robotic combined) operations. Patients that underwent open surgery had an average EBL of 576.53 mL while patients who underwent laparoscopic and robotic operations experienced significantly less EBL of 214.21 mL and 141.37 mL, respectively (*p* = 0.002, <0.001, respectively). Overall, MI operations in general displayed a statistically significantly lower average EBL of 182.20 mL as compared to open operations (*p* = 0.003). When considering blood transfusion requirements, patients who underwent open surgery required 1.19 units of blood on average. Both laparoscopic and robotic groups required significantly less blood as compared to the open approach (*p* ≤ 0.001). Additionally, in aggregate, the MI group required statistically significantly less blood transfusions, 0.09 units, on average, as compared to the open group (*p* = 0.006). In terms of days until the first bowel movement after surgery, the average for the open group was 4.40 days, while the average in the laparoscopic and robotic groups were 3.05 and 2.39, respectively (*p* = 0.029, 0.002, respectively), as compared to open operations. Overall, the minimally invasive group experienced 2.79 days until the first bowel movement after surgery which was statistically significantly less time as compared to the open group (*p* = 0.003, Table 2). Among all 3 outcomes, only EBL was significantly different between laparoscopic and robotic surgical approaches with robotic EBL being less than laparoscopic (*p* = 0.018); there were no statistically significant differences in blood transfusion requirements and days until first bowel movement between laparoscopic and robotic approaches (*p* = 0.85, 0.39, respectively, Table 3).

**Table 2:** Open short-term postoperative outcomes as compared to laparoscopic, robotic, and the combination of the two (minimally invasive).

Outcome	Surgical approach (p-value)			
	Open (reference)	Laparoscopic (p-value)	Robotic (p-value)	Minimally invasive (p-value)
Estimated Blood Loss (EBL, mL)	576.53	214.21 (0.002)	141.37 (<0.001)	182.20 (0.003)
Blood Transfusion (Units)	1.19	0.1 (<0.001)	0.08 (<0.001)	0.09 (0.006)
Days Until First Bowel Movement	4.40	3.05 (0.029)	2.39 (0.002)	2.79 (0.003)

**Table 3:** Comparison of short-term outcomes after laparoscopic and robotic approaches.

Outcome	Surgical approach		Significance
	Laparoscopic	Robotic	p-Value
Estimated blood loss (EBL, mL)	214.21	141.37	**0.018**
Blood transfusion (Units)	0.1	0.08	0.85
Days until first bowel movement	3.05	2.39	0.39

Bold data signifies that the robotic approach led to significantly less blood loss as compared to laparoscopic.

### Pathological and oncological outcomes

Oncological outcomes were assessed across all three groups. No difference in staging was identified between groups (*p* = 0.103), and all groups were able to achieve an R0 resection, adequate lymph node retrieval (≥12 lymph nodes), and adequate TME dissection (*p* = 0.856, 0.489, 0.500, respectively). Notably, significantly more patients in the robotic group underwent neoadjuvant therapy at a rate of 85.4 % as compared to those undergoing open operations of which only 55.50 % received neoadjuvant therapy (*p* = 0.008, Table 4). Average tumor size for each approach was 3.5 cm, 2.7 cm, and 2.7 cm for open, laparoscopic, and robotic operations, respectively.

**Table 4:** Oncologic outcomes across all three groups.

Outcome	Surgical approach			Significance
	Open	Laparoscopic	Robotic	p-Value
TNM stage				0.103
Stage 0	5	8	9	
Stage 1	3	2	2	
Stage 2	6	6	9	
Stage 3	9	23	14	
Stage 4	3	10	3	
Neoadjuvant	55.50 %	58.49 %	85.40 %	0.008
R0 (surgical margins)	92.9 %	92.3 %	95.1 %	0.856
Lymph node retrieval	78.60 %	79.20 %	88 %	0.489
TME	48 %	48.7 %	61.3 %	0.5

### Complications

No surgical site infection (SSI) or deep space infection (DSI) were noted for open operations. Laparoscopic operations resulted in two DSI, and robotic operations resulted in one SSI. Interestingly, the laparoscopic approach yielded the most readmissions (13). There were no significant differences found in complication rates between any of these approaches.

### Length of stay

The average length of stay (LOS) for patients who underwent MI surgery was 4.35 days which was significantly less than the LOS for patients undergoing open surgery with an LOS of 8.48 days (*p* < 0.001, Table 5). Additionally, among those undergoing MI approaches, those undergoing robotic operations tended to have shorter LOS with an average of 3.79 days as compared to 4.92 for those undergoing laparoscopic operations, although this result was not significant (*p* = 0.252, Table 6).

**Table 5:** Length of stay reported for the open approach compared to laparoscopic, robotic, and minimally invasive approaches.

Outcome	Surgical approach (p-value)			
	Open (reference)	Laparoscopic	Robotic	Minimally invasive
Length of stay (days)	8.48	4.92 (<0.001)	3.79 (<0.001)	4.35 (<0.001)

**Table 6:** Length of stay for laparoscopic compared to robotic operations.

Outcome	Surgical approach		Significance
	Laparoscopic	Robotic	p-Value
Length of stay(days)	4.92	3.79	0.252

### Survival

**Fig. 1:**
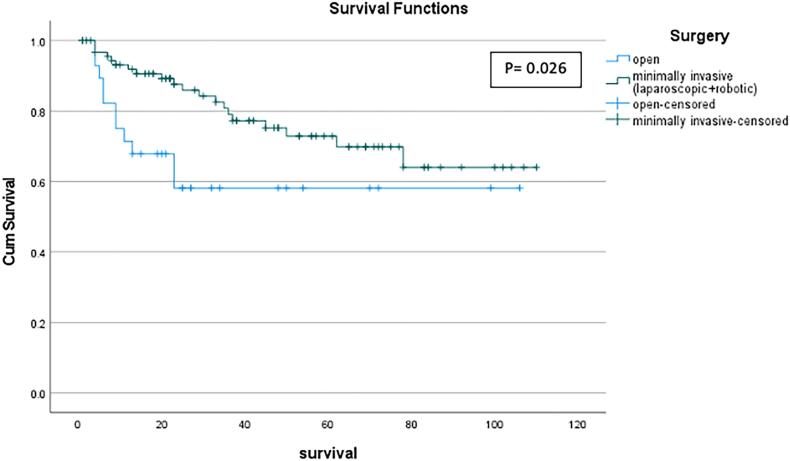
Comparison of survival rates between the open and minimally invasive approaches.

**Fig. 2:**
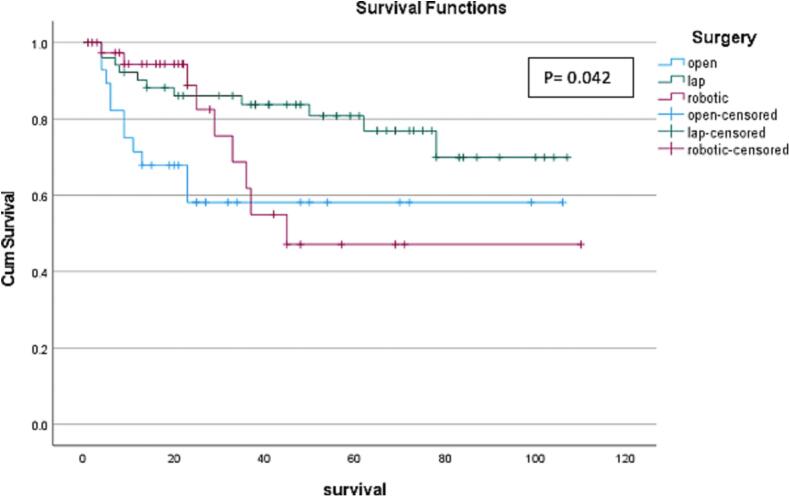
Comparison of the survival rates of all three surgical approaches.

In this study, data from 119 participants was used to compare the overall survival between MI surgery and open surgery (Fig. 1). The MI group (*n* = 91) had a mean survival of 6.97 years, while open surgery group (*n* = 28) had a significantly lower mean survival of 5.55 years (*p* = 0.026). Comparing MI operations, the laparoscopic approach had a mean survival of 7.19 years as compared to the robotic approach which had a significantly lower mean survival of 5.65 years (*p* = 0.042, Fig. 2). Interestingly, the robotic and open approaches had similar survival rates.

### Cost analysis

**Fig. 3:**
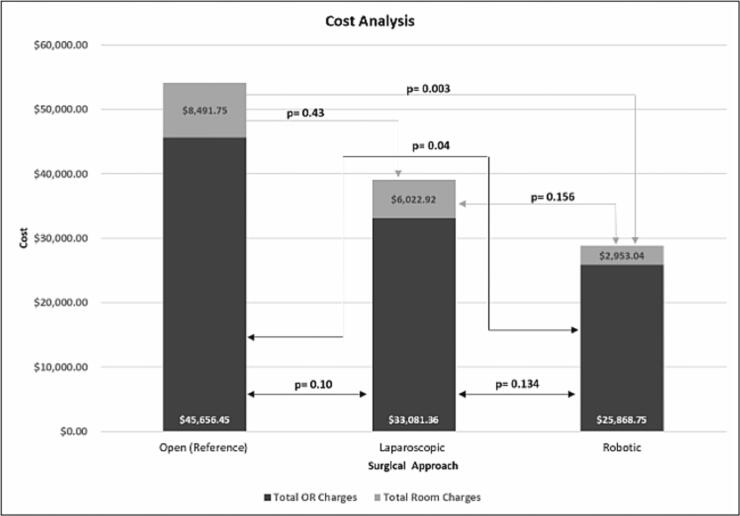
Differences in cost across all three surgical approaches.

Cost of surgery was recorded in 45 patients and compared across the approaches using average operative room (OR) charges – including OR time and supplies – and average hospital stay charges. OR charges for patients who had open surgery averaged at $45,656.45, and the average hospital stay charges were $8491.75 for a total of $54,147.45. The OR charges for patients who had laparoscopic surgery averaged at $33,081.36, and the average hospital stay charges were $6022.92 for a total of, $39,104.28, revealing that open surgery was not significantly more expensive as compared to laparoscopic (*p* = 0.10). The OR charges for robotic surgery were $25,868.75 with the average hospital stay charges adding an additional $2953.04 for a total of $28,821.79. Interestingly, this showed that at our institution, the overall charges for robotic operations were significantly less as compared to open operations, both in terms of OR and room charges (*p* = 0.04, 0.003, respectively). Additionally, although laparoscopic operations tended to be more expensive than robotic operations on average, this difference was not significant (*p* = 0.134, Fig. 3).

## Discussion and conclusion

This study focused on both short- and long-term outcomes of rectal operations over a 10-year period in the community setting. Our results showed that both robotic and laparoscopic operations resulted in lower average EBL, less blood transfusions, and less time to first bowel movement. Notably, when comparing laparoscopic and robotic operations, only EBL was significantly less with the robotic approach. This confirmed current data showing that laparoscopic and robotic operations are associated with less blood loss as compared to open operations [[6], [7], [8], [9],^12^,^13^]. In regard to time to first bowel movement, current literature does not show a difference in return of bowel function between open and MI operations [3,6,7,14,15]. The difference in our results compared to the current literature could be due to return of bowel function being a multi-factorial outcome. For this reason, without further research, it is difficult to say that this result in our study is due to causation rather than just a correlation.

We also looked at staging between groups, for which we found no significant difference; all groups were able to achieve R0 resection, adequate LN retrieval (>12 LN), and adequate TME. Current data is mixed regarding ability to achieve an R0 resection. Similar to our results, many studies have found no difference between groups with regards to R0 resection [[3], [4], [5],^16^]. However, a recent multicenter cohort study by Kethman et al. showed that open and robotic approaches were associated with a decreased likelihood of successful resection with the laparoscopic approach most likely to result in successful R0 resection [16]. Again, the literature is mixed regarding the approach that results in the best lymph node harvest while our study showed no difference between groups [2,4,15,16]. Interestingly, in our study, significantly more patients in the robotic group underwent neoadjuvant therapy as compared to open operations. As this was a retrospective study, this difference could be attributed to neoadjuvant therapy making the robotic approach feasible although this cannot be definitively determined.

Regarding length of stay, our study showed that minimally invasive operations resulted in significantly shorter LOS as compared to open. Although robotic operations tended to have shorter LOS as compared to laparoscopic, this was not significant. Our results are similar as compared to previous studies showing the laparoscopic and robotic approaches to be associated with shorter lengths of stay when compared to the open approach [13,16]. Previous studies have shown mixed results when comparing laparoscopic and robotic operations. In 3 studies, robotic operations were found to be associated with shorter length of stay [5,8,15]. In two other studies, one being a meta-analysis of RCTs, the pooled data showed no significant difference in length of stay between laparoscopic and robotic surgery [2,3]. Similarly, our study did not show a difference in LOS between laparoscopic and robotic operations. However, given the decrease in EBL and faster return of bowel function found in our study with the minimally invasive approaches, it would stand to reason that these approaches would also therefore result in a shorter LOS as compared to the open approach. Regarding complications, there was no significant difference between groups. This could be attributed to the low number of complications overall.

In terms of survival, in this study, the overall mean survival was 6.61 years across all 3 approaches. The overall 5-year survival across all groups was just under 70 %. When looking at 5-year survival in the robotic group, it is comparable to the open group; however, the number of robotic surgeries increased over the 10-year period, so many robotic operations were performed too recently to be included in the 5-year survival data at the time of this analysis. When looking at 3-year survival for our study population, the laparoscopic approach showed the best survival, followed by robotic, and then the open approach. Rationale for this difference is likely multifactorial and unable to determined by the parameters measured in this study. Current literature is mixed regarding survival in each of these groups, again highlighting that survival is related to multiple factors and that more research needs to be undertaken to determine if any of the surgical approaches can affect survival [17].

Finally, with regards to cost, our group was surprised to find that robotic operations were significantly less costly as compared to open operations. Even laparoscopic operations appeared to cost less than open operations. The difference in average total cost between open and laparoscopic operations was $12,575. Between laparoscopic and robotic approaches, the difference was $7213. This is contradictory to the current literature which would support robotic operations as being associated with the highest costs [9]. Lower cost for the robotic approach in our study could be related to the reduced length of stay as well as surgeon experience leading to cost-effective choices in the operating room and less time needed in the operating room. Additionally, this cost analysis does not include initial robot or instrument purchase which can be quite costly. Although we do not have this granularity in the data, it is likely that more staplers are used laparoscopically due to difficulty obtaining the right angle. Finally, given the well-reported costliness of robotic operations, the surgeons in this study were likely extra cautious regarding instrument selection and utility. Notably, we did not find a significant difference in cost between laparoscopic and robotic operations.

This study has several limitations. First, as it is a retrospective study, there was no randomization of patients leaving room for selection bias. An attempt was made to limit this bias by including all patients who underwent rectal operations during the selected time period. Additionally, the fact that patients who underwent robotic operations had a significantly higher rate of receiving neoadjuvant therapy could have contributed to their improved post-operative outcomes. This study also only includes a few surgeons at a single community hospital and includes a relatively small population, limiting its reproducibility and generalizability. Still, this study is an important contribution to the literature which is lacking in community-based studies.

Our findings show that minimally invasive rectal operations, especially robotic, lead to better short- and long-term outcomes, equivalent oncologic resection, and are more cost-effective as compared to open operations even in the community setting. This supports the continued performance and growth of robotic colorectal operations in the community setting, adding important findings to the literature, and importantly calls attention to the need for additional research to be done to continue to bring excellent colorectal care to communities across the country.

## CRediT authorship contribution statement

Laura Cooper aided in data analysis and writing of the final manuscript. Lena Morant and Maribeth Anderson aided in data collection and analysis and manuscript drafts. McKenzie Bedra aided in data collection and analysis. Cherif Boutros aided in final manuscript draft and oversaw all aspects of the project.

## Funding

This research did not receive any specific grant from funding agencies in the public, commercial, or not-for-profit sectors.

## Ethics approval

This study obtained institutional review board approval from the Human Research Protection Office (HP-00091709).

## Declaration of competing interest

None.
